# Genotyping‐in‐Thousands by sequencing reveals marked population structure in Western Rattlesnakes to inform conservation status

**DOI:** 10.1002/ece3.6416

**Published:** 2020-06-02

**Authors:** Danielle A. Schmidt, Purnima Govindarajulu, Karl W. Larsen, Michael A. Russello

**Affiliations:** ^1^ Department of Biology University of British Columbia Okanagan Campus Kelowna BC Canada; ^2^ British Columbia Ministry of Environment and Climate Change Strategy Victoria BC Canada; ^3^ Department of Natural Resource Sciences Thompson Rivers University Kamloops BC Canada

**Keywords:** designatable units, genotyping‐by‐sequencing, GT‐seq, hibernacula, reptile, snake

## Abstract

Delineation of units below the species level is critical for prioritizing conservation actions for species at‐risk. Genetic studies play an important role in characterizing patterns of population connectivity and diversity to inform the designation of conservation units, especially for populations that are geographically isolated. The northernmost range margin of Western Rattlesnakes (*Crotalus oreganus*) occurs in British Columbia, Canada, where it is federally classified as threatened and restricted to five geographic regions. In these areas, Western Rattlesnakes hibernate (den) communally, raising questions about connectivity within and between den complexes. At present, Western Rattlesnake conservation efforts are hindered by a complete lack of information on genetic structure and degree of isolation at multiple scales, from the den to the regional level. To fill this knowledge gap, we used Genotyping‐in‐Thousands by sequencing (GT‐seq) to genotype an optimized panel of 362 single nucleotide polymorphisms (SNPs) from individual samples (*n* = 461) collected across the snake's distribution in western Canada and neighboring Washington (USA). Hierarchical STRUCTURE analyses found evidence for population structure within and among the five geographic regions in BC, as well as in Washington. Within these regions, 11 genetically distinct complexes of dens were identified, with some regions having multiple complexes. No significant pattern of isolation‐by‐distance and generally low levels of migration were detected among den complexes across regions. Additionally, snakes within dens generally were more related than those among den complexes within a region, indicating limited movement. Overall, our results suggest that the single, recognized designatable unit for Western Rattlesnakes in Canada should be re‐assessed to proactively focus conservation efforts on preserving total genetic variation detected range‐wide. More broadly, our study demonstrates a novel application of GT‐seq for investigating patterns of diversity in wild populations at multiple scales to better inform conservation management.

## Graphical abstract

Genetic studies provide key information for the delineation of conservation units below the species level to help prioritize management efforts. Here, we used Genotyping‐in‐Thousands by sequencing (GT‐seq) to reveal significant population structure and limited connectivity among populations of the at‐risk Western Rattlesnake at their northern range periphery in Canada. Our results demonstrate a novel application of GT‐seq and suggest that the current conservation unit status for this species in Canada warrants re‐examination to better inform proactive management.

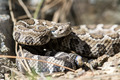

## 1 INTRODUCTION

Habitat loss and degradation are two of the main threats affecting the persistence of global biodiversity (Magin, Johnson, Groombridge, Jenkins, & Smith, 1994; Purvis, Gittleman, Cowlishaw, & Mace, 2000). Anthropogenic disturbances such as residential and commercial development, roads, agriculture, and forestry can lead to the isolation of large areas of formerly intact habitat that becomes disconnected over time. As a result, populations often are reduced in size and exhibit low connectivity and restricted gene flow (Pease, Lande, & Bull, 1989). Without sufficient genetic variation, populations of a species may be limited in their ability to adapt in the face of stochasticity, making them more vulnerable to extinction (Frankham, 2003). Over time, the extirpation of multiple isolated subpopulations can contribute to overall species decline in population size and erosion of genetic diversity. As a result, species existing in isolated populations often are of elevated conservation concern (Ehrlich, 1988).

Population genetic studies play an important role in understanding connectivity among geographically isolated populations, especially for rare or at‐risk species. In particular, genetic assessments can help delineate conservation units, which are fundamental for enforcing legislation and prioritizing conservation resources (Funk, Mckay, Hohenlohe, & Allendorf, 2012; Green, 2005). Multiple concepts for designating conservation units have been proposed within various contexts, including evolutionarily significant units (ESUs) based upon historical patterns of genetic divergence between populations (Crandall, Bininda‐Emonds, Mace, & Wayne, 2000; Ryder, 1986; Waples, 1991) and management units (MUs) that consider contemporary patterns of genetic differentiation among populations (Moritz, 1994). In Canada, designatable units (DUs) have been adopted by the Committee on the Status of Endangered Wildlife in Canada (COSEWIC) to define biologically relevant units for conservation below the species level (Green, 2005). DUs are determined based upon population discreteness and evolutionary significance, two criteria that can be examined using genetic data. According to COSEWIC, population(s) may be considered discrete if there is evidence for genetic distinctiveness, natural geographic disjunction, or occurrence in geographically varied ecological zones. Once populations are deemed discrete, they can then be evaluated for significance to determine whether they are integral to the evolutionary trajectory of the species, where unique genetic diversity would not be naturally restored if lost (Environment & Climate Change Canada, 2017). Measures of neutral genetic variation and gene flow among populations can provide evidence for discreteness, while the evolutionary significance of populations can be informed by estimating patterns of adaptive divergence (Allendorf, Hohenlohe, & Luikart, 2010; Funk et al., 2012).

**FIGURE 1:**
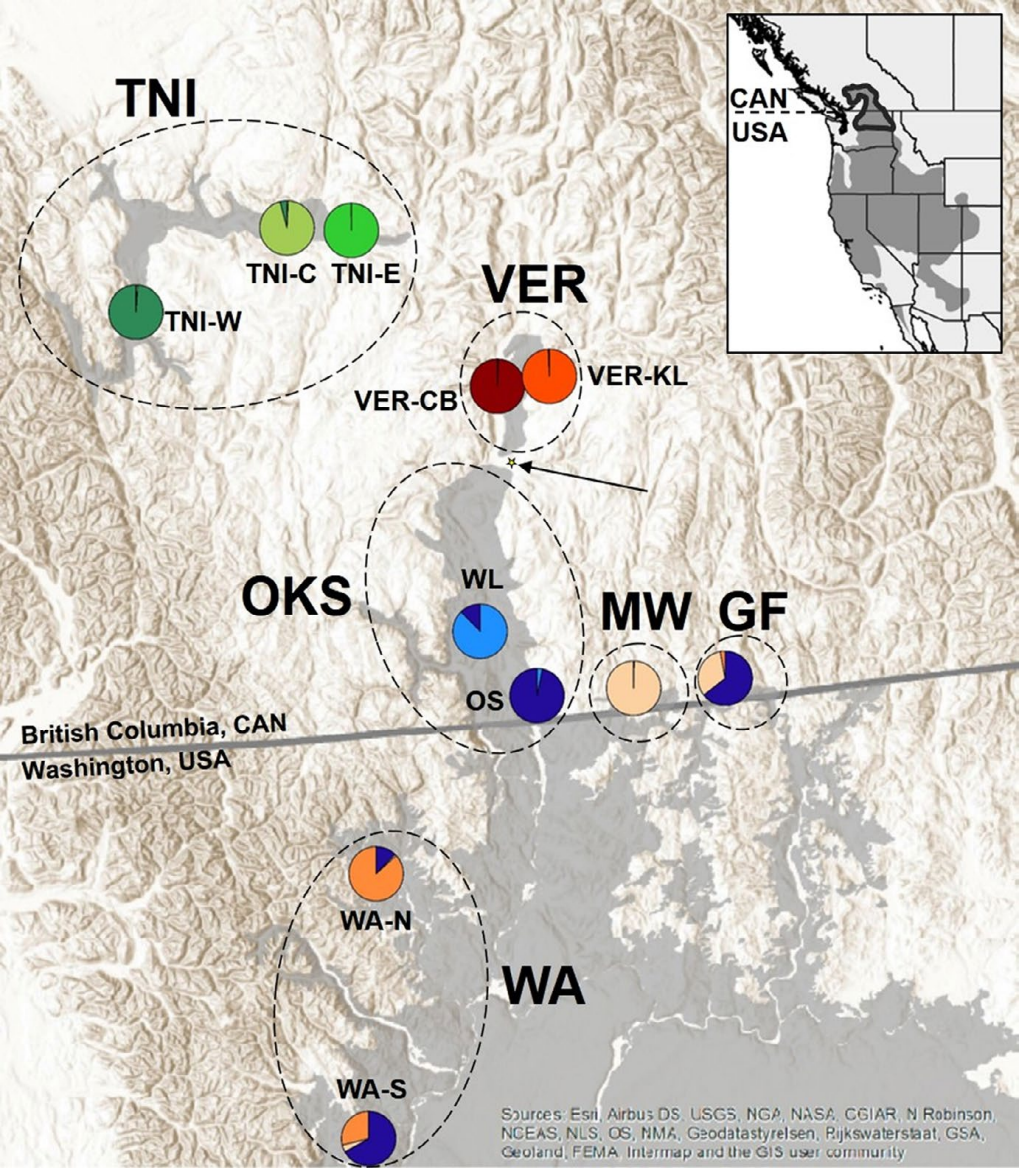
The distribution of den complex locations of Western Rattlesnakes across five main geographic regions in Canada, including Thompson‐Nicola (TNI), Vernon (VER), Okanagan‐Similkameen (OKS), Midway (MW), Grand Forks (GF), as well as Washington (WA), USA. Gray shading represents the current known distribution in Canada and Washington. Black‐dotted circles encompass geographic regions defined by COSEWIC (2015); WA was also considered a separate geographic region. Colored pie charts indicate results of the hierarchical Bayesian clustering analyses for individuals within the 11 den complexes across the six geographic regions: TNI‐W (*n* = 57), TNI‐C (*n* = 35), TNI‐E (*n* = 17), VER‐CB (*n* = 30), VER‐KL (*n* = 57), WL (*n* = 99), OS (*n* = 55), MW (*n* = 23), GF (*n* = 31), WA‐*N* (*n* = 39), WA‐S (*n* = 18). Each color within a pie chart represents the proportion of identity to each unique genetic cluster. The black arrow is pointing to the City of Kelowna, British Columbia, Canada (gray star). Gray shading in the figure inset displays the entire distribution of Western Rattlesnakes in North America with the approximate distribution in this study outlined in dark gray [spatial data from NatureServe and the International Union for the Conservation of Nature (IUCN), 2007]

One species of concern in Canada that requires an explicit evaluation of designatable unit status is the Western Rattlesnake (*Crotalus oreganus*). Although broadly distributed throughout the western half of the United States, this snake is classified as “threatened” by the Canadian Species At Risk Act and protected under the British Columbia (BC) Wildlife Act (COSEWIC, 2015). The area of occupancy of this species in Canada is restricted to BC and estimated at <1,500 km^2^ (COSEWIC, 2015; Figure 1), representing one of the northernmost extensions for any viper in North America (Family Viperidae). At these northern latitudes, Western Rattlesnakes use traditional denning sites that enable overwinter survival (Charland, Nelson, & Gregory, 1993). The numbers of animals using these dens range from single digits to several hundred individuals, including adults and juveniles of both sexes (Winton, Taylor, Bishop, & Larsen, 2018). Following emergence from the dens in the spring, adult snakes migrate various distances to forage and mate before returning to the same dens in the fall (Gomez, Larsen, & Gregory, 2015; Harvey & Larsen, in press). Gravid females do not travel as far, remaining closer or even at den sites to give birth to their litters in late summer (Eye and Larsen unpubl.). Presently, the entire distribution of the Western Rattlesnake in Canada is managed as a single DU (COSEWIC, 2015), although five putative geographic regions have been identified (Figure 1). These regions are reasonably distinct, given that suitable habitat at this latitude is relatively scarce and naturally interrupted by geographic barriers. Moreover, anthropogenic modifications to the landscape, such as habitat loss and fragmentation by roads, likely constitute additional barriers to gene flow in this species (Maida et al., 2018; Winton et al., 2018).

In addition to the influence of natural and anthropogenic barriers to gene flow, range position can impact the extent and distribution of population genetic variation in broadly distributed species. The central–marginal hypothesis (Eckert, Samis, & Lougheed, 2008) predicts that peripheral populations, particularly those in discontinuous patches, are expected to have lower genetic diversity within and greater differentiation among populations than those found toward the core of a species range. Despite smaller population sizes and an increased risk of extirpation, these peripheral populations may also have high evolutionary potential given they are likely subject to more heterogeneous environments than found in the range core (Cassel‐Lundhagen et al., 2009; Lesica & Allendorf, 1995). Such conditions may lead to the development of unique traits and divergent genetic variation within peripheral populations that permit adaptation to environmental change (Lesica & Allendorf, 1995). For Western Rattlesnakes, a fuller understanding of the distribution of genetic variation within and among populations at the northernmost extent of their species range is warranted for evaluating discreteness, inferring significance, and refining conservation strategies.

In the current study, we used genotyping‐by‐sequencing (GBS) to characterize the distribution of genetic variation within and among Western Rattlesnake populations in Canada and neighboring Washington (USA). Given the protected status and venomous nature of the animal, we largely employed minimally invasive sampling (MIS) using cloacal swabs, as it reduced handling time and limited disturbance to individual snakes. Although MIS is often suboptimal for use with many GBS approaches (e.g., RADseq; Andrews, Barba, Russello, & Waits, 2018; Russello, Waterhouse, Etter, & Johnson, 2015), we recently demonstrated the utility of a multiplex amplicon sequencing approach, Genotyping‐in‐Thousands by sequencing (GT‐seq; Campbell, Harmon, & Narum, 2015), for cloacal swab samples and other MIS sources (e.g., skin sheds) for the Western Rattlesnake (Schmidt, Campbell, Govindarajulu, Larsen, & Russello, 2020). Here, we used genotypic data collected from 591 individuals to infer patterns of hierarchical genetic structure within and among the five presumed geographic regions of Western Rattlesnakes in British Columbia (Canada) as well as in Washington (USA). We also examined fine‐scale patterns of genetic diversity and connectivity among complexes of dens within regions. Lastly, we used the evidence collected to explicitly address DUs for Western Rattlesnakes in Canada and discuss the implications of these results for informing prioritization and management strategies.

## 2 METHODS

### 2.1 Study area and sample collection

Between 2015 and 2017, a total of 591 individuals were sampled, including from blood (*n* = 30), cloacal swabs (*n* = 552), buccal swabs (*n* = 5), and skin sheds (*n* = 4), from 36 dens across all five of the informally recognized regions of the Western Rattlesnake range in Canada, namely the Thompson‐Nicola (TNI), Vernon (VER), Okanagan‐Similkameen (OKS), Midway (MW), and Grand Forks (GF), as well as from neighboring Washington (WA), USA (Figure 1).

All DNA samples obtained from live snakes were taken with the anterior portion of the animals restrained in a plexiglass handling tube (Murphy, 1971). Blood samples were collected by inserting a sterile syringe into the caudal vein of a snake, drawing 0.1 ml of blood, and then directly transferring the sample to 1 ml of Longmire lysis buffer (Longmire, Maltbie, & Baker, 1997) for preservation at 4°C. Cloacal samples were collected by gently inserting a sterile cotton‐tipped swab into the cloacal vent, located on the ventral side of the snake near the tail, and then slowly rotating it 5–10 times. For juvenile and neonate snakes that were too small for cloacal sampling, we employed buccal swabs. To collect buccal cells, a cotton swab was gently inserted into the mouth of a snake and slowly rotated for 5–10 s, with care taken to avoid getting the swab stuck on the fangs. As with blood samples, buccal and cloacal swabs were then preserved in 1 ml of Longmire lysis buffer (Longmire et al., 1997) at 4°C. Shed skins were stored at −80°C as recommended by Burbrink and Castoe (2009).

In addition to collecting DNA samples, we also recorded the snout‐to‐vent length (SVL), sex, weight (g), and age class for individual snakes when possible. For the present study, we defined adults as those snakes with a SVL measurement of >540 mm either at the time of sampling or postsampling, based upon recent estimates for sexually mature Western Rattlesnakes (Maida et al., 2018). All snakes were marked with a unique colored marking at the base of the rattle to identify recaptured individuals both within and among sampling years. Sample collection took place under Thompson Rivers University Animal Ethics File No. 101547, BC Ministry of Environment Park Use Permit No. 108794, and British Columbia Wildlife Act PE15‐171661.

### 2.2 DNA extraction

We extracted whole genomic DNA from the blood samples using a DNeasy Blood and Tissue Kit (Qiagen) following the manufacturer's protocols (including RNAse A treatment). Only slight variations were applied for the shed, buccal, and cloacal swab samples. For the shed skins, ~160–180 mg of each shed underwent a surface clean with a 3% bleach solution. After bleach treatment, we then washed the sheds with ethanol and left them to air‐dry completely. Once dry, we manually pulverized each sample and placed them into separate 2‐ml tubes. Proteinase K and lysis buffer were added to each tube, and samples were incubated for 48 hr at 56°C. The rest of the extraction procedure followed the manufacturer's protocol (including RNAse A treatment).

For the cloacal and buccal swab samples, we extracted DNA from the lysis buffer in which the swabs were stored. 180 μl of buffer from each sample was incubated with 20 μl of Proteinase *K* for 1 hr at 56°C. The rest of the extractions then followed the manufacturer's protocol (including RNAse A treatment). Once eluted, we quantified all DNA extracts using a Qubit™ 3.0 Fluorometer and Qubit™ dsDNA High Sensitivity Assay kit (Invitrogen).

### 2.3 GT‐Seq library preparation and genotyping

We prepared a GT‐seq library to simultaneously genotype all 591 samples at a targeted panel of 362 single nucleotide polymorphism (SNP) markers previously developed and optimized in Schmidt et al. (2020). A total of 17 samples were repeated to calculate within library genotyping error. Library preparation generally followed the protocol of Campbell et al. (2015) as modified in Schmidt et al. (2020). For PCR1, we ran one plate per primer pool to amplify the specific regions of DNA containing SNPs of interest within each individual. Next, 3 μl of each resulting PCR product was pooled together and diluted 1:10 (a total dilution of 1:20) for use in PCR2 to add identifying barcodes for each individual sample and the corresponding sample location in each PCR plate. After each plate of 96 PCR2 products was normalized using a SequelPrep Normalization kit (Thermo Fisher), we pooled 10 μL of sample from each individual into a plate library. Plate libraries were then quantified, and an equimolar amount of each was combined in a single tube. We purified this library using a MinElute PCR Purification Kit (Qiagen) eluted in a final volume of 22 μl. Library sequencing was completed on a single lane of Illumina HiSeq 4000 paired‐end 100 sequencing at McGill University and Génome Québec Innovation Centre. Raw sequencing data were genotyped using the GT‐seq pipeline available on GitHub (https://github.com/GTseq/GTseq‐Pipeline). The *GTseq_Genotyper_v3.pl* script was used to call genotypes for each individual sample, and then, *GTseq_GenoCompile_v3.pl* was used to compile all the individual genotypes. This resulting output file was then converted into a PED file format (Purcell et al., 2007) for downstream genetic analysis.

Prior to analysis, we quality‐filtered the entire dataset of 608 samples (591 individuals + 17 repeats). Those individuals and SNP loci with >30% missing data were removed from the dataset. Loci with a minor allele frequency <0.05 and those that deviated from Hardy–Weinberg Equilibrium (HWE) in >60% of sampling units were also removed. We calculated genotyping error for repeated samples that passed data quality filters (*n* = 17) as the proportion of discordant genotypes across all loci successfully genotyped for both replicates. To reduce bias due to sampling potentially highly related individuals at dens, we used ML‐RELATE (Kalinowski, Wagner, & Taper, 2006) to infer relationships between all individuals using default parameters. After cross‐referencing with the age class data, those individuals with an inferred parent as a result of this analysis were subsequently removed from the dataset for all population genetic analyses, except those assessing patterns of relatedness.

### 2.4 Population structure and migration

To infer broad‐scale patterns of population structure across the northern range of the Western Rattlesnake, we conducted Bayesian clustering analyses with STRUCTURE v 2.3.4 (Pritchard, Stephens, & Donnelly, 2000). Ten iterations were run for *K* = 1–8 (the number of a priori geographic regions + 2), each with a burn‐in of 500,000 and a run length of 1,000,000 MCMC steps. The resulting output was then summarized using STRUCTURE HARVESTER (Earl & vonHoldt, 2012). To infer the optimal *K* value, we employed a combination of the Δ*K* method (Evanno, Regnaut, & Goudet, 2005) and the plotting of the log probability of the data (Pritchard et al., 2000) to assess where ln Pr(X|K) plateaued (see STRUCTURE manual). Additional iterations of STRUCTURE were also conducted for each independent cluster to test for fine‐scale genetic substructure. For the optimal values of *K*, results were summarized with CLUMPP (Jakobsson & Rosenberg, 2007) and then plotted with DISTRUCT v.1.1 (Rosenberg, 2004). Based on these analyses, we defined those groups of dens that either formed distinct genetic clusters or were geographically isolated as “den complexes” (Figure 1). Using this information, we calculated Weir and Cockerham's (1984) unbiased estimates of pairwise genetic differentiation (*θ*) among sampling regions and den complexes as implemented in GENETIX 4.05 (Belkhir, Borsa, Chikhi, Raufaste, & Bonhomme, 2004).

To further examine connectivity across the range, we tested for patterns of isolation‐by‐distance (Wright, 1943) among all den complexes using a Mantel test (Mantel, 1967) as implemented in the R package *ade4* (Dray & Dufour, 2007) using 10,000 replicates to test for significance. To conduct this test, the centroid location of each den complex was determined using the *rgeos* (Bivand & Rundel, 2014) package in R (R Core Team, 2019). The straight‐line geographic distance between centroids was then calculated in kilometers with GeographicDistanceMatrixGenerator_v1.2.3 (http://biodiversityinformatics.amnh.org/open_source/gdmg/download.php) and correlated with estimates of pairwise *θ*. We then repeated this test using den‐level pairwise geographic and genetic distances within each geographic region containing >2 dens to evaluate more fine‐scale patterns of connectivity.

We also assessed the amount and direction of contemporary migration among den complexes with BAYESASS 3.04 (Wilson & Rannala, 2003). This Bayesian approach uses multilocus genotypes to estimate movement among populations over the past three generations. We estimated levels of migration using 10,000,000 iterations after a burn‐in of 1,000,000 MCMC steps, sampling after every 100 steps. Five runs were conducted, each starting with a different random seed, and convergence was analyzed by comparing mean posterior estimates of migration. To determine whether migration between den complexes was significant, we calculated 95% credible sets by calculating the mean migration rate ±1.96x the standard deviation, as suggested in the user manual. Those migration estimates with credible sets that did not include zero were deemed significant.

### 2.5 Genetic diversity and relatedness within and among den complexes

We evaluated genetic diversity within each den complex and within each den by calculating estimates of observed heterozygosity (*H*_o_), expected heterozygosity (*H*_E_), nucleotide diversity (π), and the proportion of variable sites (*P*) as implemented in ARLEQUIN (Excoffier & Lischer, 2010). Global tests for heterozygote deficit or excess were run using Fisher's exact tests in GENEPOP 4.5 (Rousset, 2008), with 10,000 dememorization steps, 100 batches, and 10,000 iterations. Those groups with a *p*‐value <.05 were considered to significantly deviate from the level of heterozygosity expected if they were in Hardy–Weinberg equilibrium. We also calculated inbreeding coefficients (*F*_IS_) for each den complex with GENETIX (Belkhir et al., 2004), using 1,000 permutations to test for significance. To determine whether measures of genetic diversity were correlated with latitude, linear regressions with nucleotide diversity and the proportion of polymorphic SNP loci (*P*) in a den complex were conducted in R (R Core Team, 2019) for all 11 den complexes.

Additionally, pairwise estimates of Queller and Goodnight's unbiased relatedness coefficient were calculated for individuals across each geographic region as well as within and among den complexes and dens using SPAGeDI 1.5 (Hardy & Vekemans, 2002). Reference allele frequencies were determined by pooling all individuals in a given geographic sampling region. For this analysis, we added the individuals with presumed parents back into the dataset.

## 3 RESULTS

### 3.1 GT‐seq genotyping

A total of 608 samples (591 individuals + 17 repeats) were genotyped, with an average of 341,838 raw sequencing reads per sample. After quality filtering for 30% missing data, minor allele frequency > 0.05, and HWE, 505 samples and 308 loci remained in our dataset. Mean genotyping error across all repeated samples (*n* = 17) was 0.36%. After repeated samples and those individuals with an inferred parent in the dataset (*n* = 27) were removed, a total of 461 individuals were used for subsequent population genetic analysis with an average depth of 332x (range: 23x‐1113x) and 3.82% missing data (range: 0.00%–29.87%).

### 3.2 Population structure and connectivity

**FIGURE 2:**
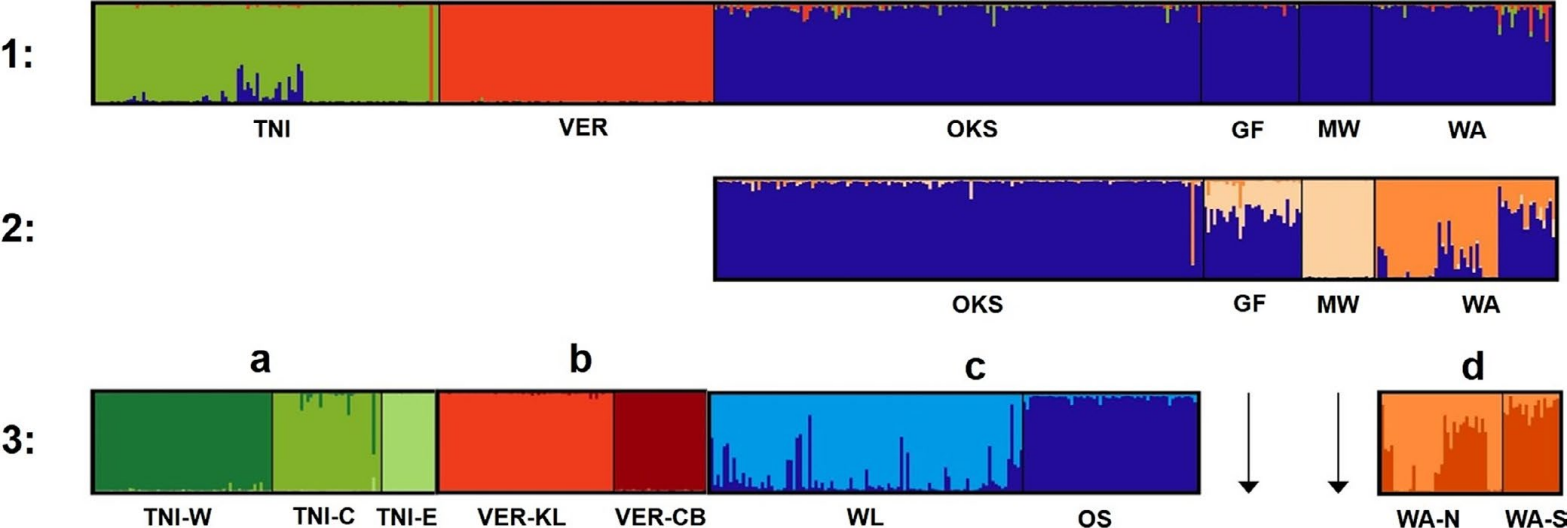
STRUCTURE bar plots displaying inferred clustering and ancestry estimation for all individuals (*n* = 461), showing *K* = 3 for iteration 1 and *K* = 3 for iteration 2 for analyses run among geographic regions. Iteration 3 investigated evidence for further substructure within regions, revealing *K* = 3, *K* = 2, *K* = 2, and *K* = 2, respectively (population acronyms below plots as defined in Figure 1). Each color represents a unique genetic cluster, and each vertical bar represents the proportion of ancestry to each cluster for each individual. For iteration 3, each letter (a, b, c, d) corresponds to a different STRUCTURE analysis. See Appendix (Figure A1) for the data used to determine optimal *K* values

**FIGURE A1:**
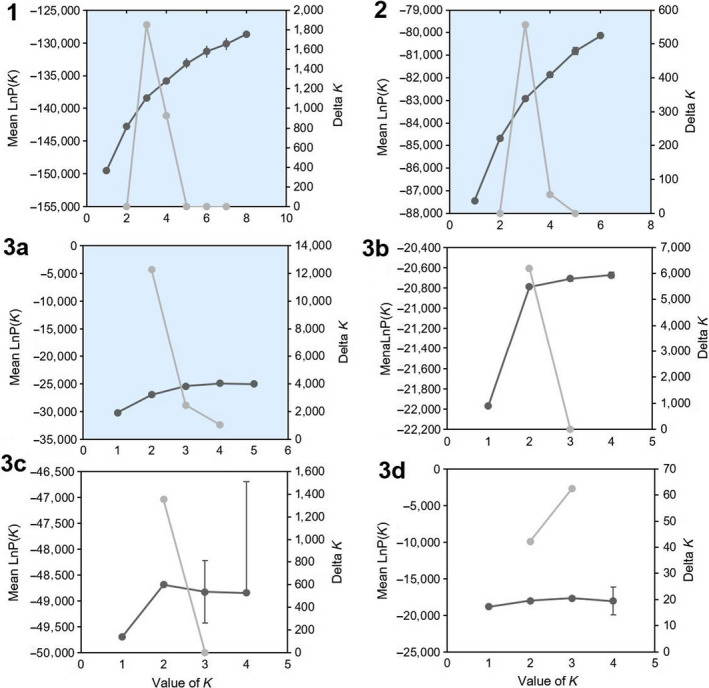
Line graphs depicting results of all iterations of STRUCTURE. Plot numbers correspond to the labels defined for each iteration of STRUCTURE in Figure 2. Dark gray lines represent the mean log probability for each *K* value (Mean ln*P*(*K*)) with vertical bars to show standard deviation. Light gray lines represent the value of Δ*K* across *K* values. Graphs with a blue background were determined to have an optimal *K* value of 3, while those with no colored background were determined to have an optimal *K* value of 2

STRUCTURE analyses inferred an initial optimal value of *K* = 3 for all samples range‐wide (Figure 2), including clusters representing TNI, VER, and the rest of the geographic regions across the range, respectively (acronyms as defined in Figure 1). Within the three genetically distinct clusters inferred range‐wide, hierarchical STRUCTURE analyses identified genetic substructure within the TNI, VER, OKS, and WA regions, a unique cluster in MW, and a geographically isolated cluster in GF that shared ancestry with both OKS and MW (Figures 1 and 2, Figure A1). Three distinct den complexes (*K* = 3) were detected in the TNI region (TNI‐W, TNI‐C, and TNI‐E) (Figure 2), while two discrete den complexes (*K* = 2) were found within each of the VER (VER‐KL, VER‐CB), OKS (WL, OS), and WA (WA‐N, WA‐S) regions, respectively, for a total of 11 genetically unique or geographically isolated den complexes. Among sampling regions, all values of pairwise genetic differentiation (*θ*) were significant (*p* < .001) and ranged from 0.059 to 0.321 (Table 1). Across the identified den complexes, estimates of *θ* ranged from 0.0459 to 0.526 (*p* < .001) (Table 2).

**TABLE 1:** Estimates of pairwise genetic distance (*θ*) between all five geographic regions in Canada, including Thompson‐Nicola (TNI), Vernon (VER), Okanagan‐Similkameen (OKS), Midway (MW), Grand Forks (GF), as well as Washington (WA), USA. All comparisons were significant (*p* < .001) with 1,000 replicates

	OKS	WA	TNI	VER	GF	MW
OKS	—	0.059	0.091	0.148	0.082	0.183
WA		—	0.114	0.172	0.105	0.202
TNI			—	0.213	0.163	0.261
VER				—	0.210	0.321
GF					—	0.199
MW						—

**TABLE 2:** Estimates of pairwise *θ* and straight‐line distance (km) between the 11 defined den complexes in this study (abbreviations as in Figure 1)

	OS	WA‐*N*	WA‐S	WL	TNI‐C	TNI‐W	TNI‐E	VER‐KL	VER‐CB	GF	MW
OS	—	0.088	0.063	0.046	0.168	0.117	0.307	0.159	0.186	0.094	0.216
WA‐N	80.1	—	0.069	0.092	0.219	0.143	0.342	0.210	0.230	0.127	0.232
WA‐S	238.3	174.5	—	0.068	0.180	0.115	0.367	0.174	0.196	0.115	0.213
WL	37.8	95.4	265.5	—	0.160	0.111	0.296	0.175	0.197	0.096	0.191
TNI‐C	200.3	238.9	412.4	162.9	—	0.124	0.405	0.287	0.308	0.242	0.352
TNI‐W	213.9	231.3	397.7	176.7	62.3	—	0.346	0.249	0.263	0.178	0.276
TNI‐E	186.7	232.1	406.4	150.1	23.5	82.9	—	0.424	0.474	0.387	0.526
VER‐KL	129.6	196.8	366.4	101.7	107.9	155.4	85.5	—	0.116	0.219	0.336
VER‐CB	122.0	185.6	356.9	91.4	100.4	143.8	79.3	15.4	—	0.252	0.372
GF	73.7	145.0	272.6	96.9	235.8	264.7	216.9	138.2	138.9	—	0.199
MW	37.8	112.0	253.8	64.5	216.2	238.5	199.5	129.0	125.7	35.9	—

Values above the diagonal represent *θ* and those below are straight‐line distances. All pairwise comparisons of *θ* were significant *p* < .001 with 1,000 replicates

**FIGURE 3:**
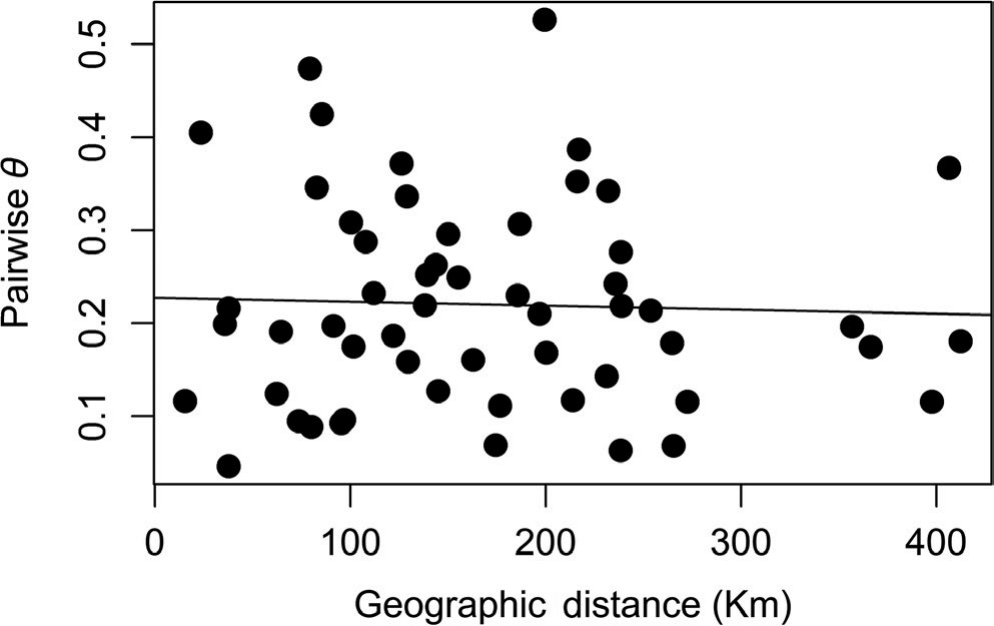
Mantel's correlation between pairwise straight‐line distance (km) and genetic distance (*θ*) for all 11 den complexes (*r* = −.03812, *p* = .4989)

Range‐wide, there was no detected pattern of isolation‐by‐distance among den complexes (*r* = −.038, *p* = .50) (Figure 3). Within sampling regions, there was no significant pattern of isolation‐by‐distance detected among dens within the TNI (*r* = .221, *p* = .298) or GF (*r* = .911, *p* = .167) regions; however, a significant pattern of isolation‐by‐distance was detected among dens in VER (*r* = .873, *p* = .03), OKS (*r* = .811, *p* < .001), and WA (*r* = .57, *p* < .001). Estimates of recent migration found significant gene flow from the OS den complex to WA‐S and from OS to WL. All other estimates of gene flow were low (*m* < 0.1) and not significant (Table 3).

**TABLE 3:** Estimates of contemporary migration among den complexes as calculated in BAYESASS. Numbers represent the mean proportion of migrants moving from one group to another per generation averaged across five iterations

		From										
		OS	WA‐*N*	WA‐S	WL	TNI‐C	TNI‐W	TNI‐E	VER‐KL	VER‐CB	GF	MW
To	OS	*0.949*	0.005	0.005	0.005	0.005	0.005	0.005	0.005	0.005	0.005	0.005
	WA‐N	0.042	*0.888*	0.013	0.010	0.007	0.007	0.007	0.007	0.007	0.007	0.007
	WA‐S	**0.216**	0.012	*0.678*	0.014	0.012	0.012	0.011	0.011	0.012	0.011	0.012
	WL	**0.065**	0.006	0.003	*0.904*	0.003	0.003	0.003	0.003	0.003	0.003	0.003
	TNI‐C	0.007	0.007	0.007	0.007	*0.917*	0.011	0.007	0.007	0.015	0.007	0.007
	TNI‐W	0.005	0.005	0.005	0.005	0.005	*0.951*	0.005	0.005	0.005	0.005	0.005
	TNI‐E	0.012	0.012	0.012	0.012	0.012	0.012	*0.881*	0.012	0.012	0.012	0.012
	VER‐KL	0.005	0.005	0.005	0.005	0.005	0.005	0.005	*0.951*	0.005	0.005	0.005
	VER‐CB	0.008	0.008	0.008	0.008	0.008	0.008	0.008	0.009	*0.918*	0.008	0.008
	GF	0.008	0.008	0.008	0.008	0.008	0.008	0.008	0.008	0.008	*0.921*	0.008
	MW	0.010	0.010	0.010	0.010	0.010	0.010	0.010	0.010	0.010	0.010	*0.902*

Italicized values along the diagonal indicate the mean proportion of nonmigrants within a den complex. Bolded values indicate significant estimates of migration where calculated 95% credible sets did not include zero.

### 3.3 Genetic diversity and relatedness

**FIGURE 4:**
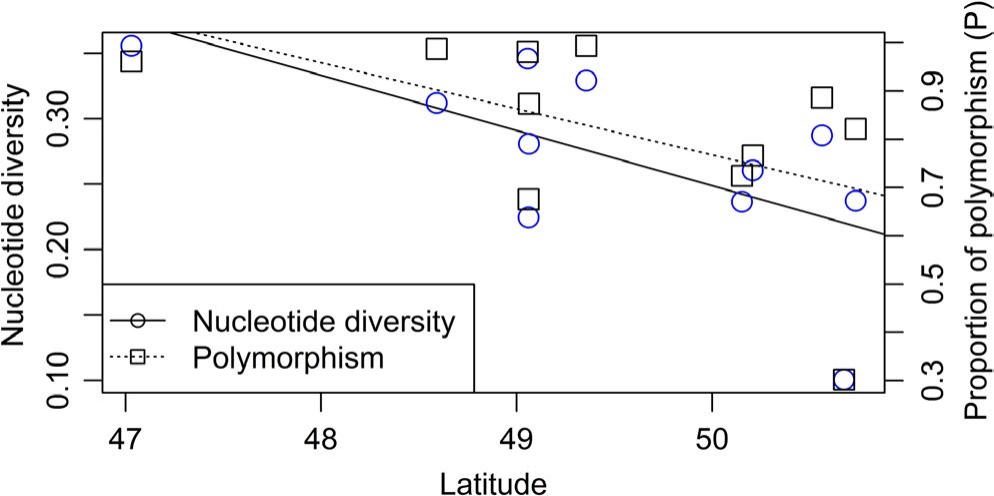
Correlation between latitude and nucleotide diversity (*π*) (*r* ^2^ = .365, *p* = .03), and latitude and proportion of polymorphism (*P*) (*r* ^2^ = .196, *p* = .1) across all 11 den complexes

Observed values of heterozygosity varied slightly across the range, with the highest value in the VER‐CB den complex (*H*_o_ = 0.379) and the lowest in the TNI‐C den complex (*H*_o_ = 0.290) (Table 4). A significant heterozygote deficit was found in the WA‐N, WA‐S, WL, TNI‐C, and TNI‐W den complexes, all accompanied by inbreeding coefficients significantly above zero (range: 0.0219–0.102). The VER‐KL den complex also exhibited a significant level of inbreeding (*F*_IS_ = 0.017) (Table 4), while a significant heterozygote excess was found in the VER‐CB group. Across den complexes, the proportion of polymorphic sites within each group varied widely. The largest proportion of polymorphism was found in the WL den complex (*p* = .994), while the least polymorphism was found in the TNI‐E den complex (*p* = .302) (Table 4). There was no significant correlation detected between latitude and proportion of polymorphism (*r* ^2^ = .196, *p* = .10); however, nucleotide diversity was significantly and negatively correlated with latitude (*r* ^2^ = .365, *p* = .03) (Figure 4).

**TABLE 4:** Estimates of mean observed and expected heterozygosity, proportion of polymorphism (P ), nucleotide diversity (π ), and inbreeding coefficients (F IS) for each den complex and dens within each den complex. * indicates *p* < .05, ** indicates *p* < .001

Den complex	Den	*n*	*P*	Mean *H*_o_	Mean *H*_E_	*F*_IS_	*π*
OS		55	0.981	0.362	0.360	−0.005	0.346
	OS‐01	28	0.974	0.365	0.360	−0.014	0.344
	OS‐02	8	0.909	0.378	0.382	0.011	0.344
	OS‐03	19	0.961	0.376	0.371	−0.012	0.341
WA‐N		39	0.987	0.312*	0.346	0.102**	0.312
	WA‐N01	3	0.682	0.452	0.468	0.054	0.337
	WA‐N02	1	0.312	1.000	1.000	0.000	0.314
	WA‐N03	9	0.724	0.352*	0.375	0.084*	0.264
	WA‐N04	9	0.802	0.353**	0.368	0.043*	0.281
	WA‐N05	3	0.494	0.454	0.454	−0.025	0.319
	WA‐N06	5	0.779	0.442*	0.418	−0.071	0.293
	WA‐N07	9	0.782	0.369	0.380	0.027	0.285
WA‐S		18	0.961	0.363*	0.375	0.031*	0.356
	WA‐S01	2	0.630	0.603*	0.564	−0.109	0.357
	WA‐S02	11	0.919	0.392	0.390	−0.005	0.355
	WA‐S03	4	0.701	0.456	0.441	−0.028	0.307
	WA‐S04	1	0.276	1.000	1.000	0.000	0.278
WL		99	0.994	0.349*	0.357	0.022**	0.329
	WL‐01	28	0.971	0.357	0.362	0.012	0.334
	WL‐02	26	0.961	0.360	0.357	−0.007	0.325
	WL‐03	21	0.932	0.364	0.361	−0.002	0.308
	WL‐04	2	0.646	0.550	0.555	0.013	0.370
	WL‐05	20	0.948	0.354**	0.370	0.046*	0.337
	WL‐06	2	0.604	0.554	0.552	−0.005	0.343
TNI‐W		57	0.886	0.331*	0.341	0.026**	0.287
	TNI‐W01	36	0.844	0.340	0.337	−0.010	0.278
	TNI‐W02	21	0.802	0.367	0.361	−0.014	0.282
TNI‐C		35	0.821	0.290**	0.307	0.062**	0.237
	TNI‐C01	9	0.545	0.365	0.359	0.004	0.209
	TNI‐C02	9	0.779	0.305**	0.340	0.111*	0.260
	TNI‐C03	17	0.744	0.344	0.344	0.003	0.232
TNI‐E	TNI‐E01	17	0.302	0.363	0.353	−0.033	0.100
VER‐KL		57	0.766	0.338	0.343	0.017*	0.260
	VER‐KL01	17	0.724	0.349	0.351	0.003	0.251
	VER‐KL02	23	0.750	0.348	0.343	−0.013	0.259
	VER‐KL03	3	0.536	0.499*	0.465	−0.078	0.252
	VER‐KL04	14	0.747	0.360	0.367	0.018	0.256
VER‐CB		30	0.724	0.379*	0.353	−0.070	0.237
	VER‐CB01	17	0.685	0.401**	0.376	−0.063	0.222
	VER‐CB02	13	0.682	0.390**	0.351	−0.109	0.244
GF		31	0.873	0.335*	0.341	0.021	0.281
	GF‐01	12	0.776	0.361	0.356	−0.020	0.257
	GF‐02	18	0.857	0.346	0.355	0.024	0.296
	GF‐03	1	0.263	1.000	1.000	0.000	0.281
MW	MW‐01	23	0.675	0.349	0.350	0.002	0.225

Note

*n* refers to the number of individuals within a specified den complex or den site.

At the individual den level, estimates of heterozygosity tended to be higher than those calculated at the den complex level (Table 4). Values of observed heterozygosity ranged from 0.305 (TNI‐C02) to 0.603 (WA‐S01). Evidence for a significant heterozygote deficit was found in the WA‐N03, WA‐N04, WL‐05, and TNI‐C02 dens, all of which also exhibited significant levels of inbreeding (Table 4). In contrast, the WA‐N06, WA‐S01, VER‐KL03, VER‐CB01, and VER‐CB02 dens all displayed an excess of heterozygotes, which was not observed in their associated den complexes overall (WA‐N, WA‐S, VER‐KL, or VER‐CB). Estimates of nucleotide diversity and the proportion of polymorphism among individual dens were similar to those determined at the den complex level (Table 4).

Additionally, average pairwise relatedness generally was higher between snakes within a den than either among dens in a den complex or among all snakes within a geographic region, though this varied across the distribution. Across regions, mean pairwise relatedness ranged from −0.05 in MW to −0.005 in OKS (Table 5). TNI‐E was the most related of all 11 den complexes (*r*_QG_ = .781), while MW was the least related (*r*_QG_ = −.047) (Table 5). Within dens, mean pairwise relatedness varied from −0.077 (WL‐04) to 0.781 (TNI‐E01) (Table 5).

**TABLE 5:** Estimates of Queller and Goodnight's (1989) pairwise relatedness (*r*_QG_) between pairs of individuals within each region, den complex, and each den

Region	Den complex	Den	*n*	*r*_QG_
TNI			112	−0.010
	TNI‐W		59	0.143
		TNI‐W01	36	0.147
		TNI‐W02	23	0.133
	TNI‐C		36	0.268
		TNI‐C01	9	0.409
		TNI‐C02	18	0.247
		TNI‐C03	9	0.219
	TNI‐E	TNI‐E01	17	0.781
VER			101	−0.010
	VER‐CB		38	0.184
		VER‐CB01	13	0.236
		VER‐CB02	25	0.191
	VER‐KL		63	0.067
		VER‐KL01	21	0.121
		VER‐KL02	24	0.087
		VER‐KL03	3	0.250
		VER‐KL04	15	0.076
OKS			162	−0.006
	OS		57	0.054
		OS‐01	30	0.068
		OS‐02	8	0.069
		OS‐03	19	0.056
	WL		105	0.028
		WL‐01	28	0.023
		WL‐02	26	0.065
		WL‐03	22	0.089
		WL‐04	2	−0.077
		WL‐05	25	0.037
		WL‐06	2	0.078
WA			58	−0.017
	WA‐N		40	0.050
		WA‐N01	3	0.077
		WA‐N02	1	‐‐‐‐
		WA‐N03	10	0.235
		WA‐N04	9	0.202
		WA‐N05	3	0.119
		WA‐N06	5	0.135
		WA‐N07	9	0.228
	WA‐S		18	0.030
		WA‐S01	2	0.142
		WA‐S02	11	0.055
		WA‐S03	4	0.253
		WA‐S04	1	‐‐‐‐
GF	GF		32	−0.033
		GF‐01	12	0.044
		GF‐02	19	−0.047
		GF‐03	1	‐‐‐‐
MW	MW	MW‐01	23	−0.047

Note

*n* refers to the number of individuals within a specified region, den complex, or den site.

## 4 DISCUSSION

### 4.1 Population structure and connectivity

We found evidence for significant hierarchical genetic structure and low connectivity among Western Rattlesnakes at the northern extent of their range in Canada and Washington (USA). At the broad scale, the five main geographic regions (TNI, VER, OKS, MW, and GF) in Canada represented three unique genetic clusters (Figure 2). In particular, TNI and VER each represented a unique genetic cluster and exhibited a high degree of genetic differentiation from all other sampled regions (Figure 2, Table 1). The other geographic regions (OKS, MW, and GF) together represented a third unique genetic cluster, with evidence for underlying substructure where OKS and MW were found to be genetically distinct (Figure 2, Table 1). In the case of GF, shared ancestry was detected with both OKS and MW, yet this region was still significantly differentiated from both based on pairwise *θ* (Table 1). Overall, the broad‐scale patterns of genetic distinctiveness identified across the distribution generally align with identified geographic disjunction between regions due to the presence of unsuitable habitat (COSEWIC, 2015). This is especially apparent between the TNI and VER regions, where there is a high and significant level of genetic differentiation (Table 1) that corresponds to a large disconnect in the species range (Figure 1). This separation is believed to have existed since the Holocene Climate Optimum (Hobbs, 2013), after which temperatures began to cool roughly 6,000–5,000 years before present representing the start of a period known as the “Mesothermic Interval” in BC (Walker, 2004).

At a finer scale, we found evidence for low and generally nonsignificant estimates of genetic migration, with no significant pattern of isolation‐by‐distance among all 11 den complexes that we defined across the distribution (Table 3, Figure 3). While male Western Rattlesnakes will move considerable distances during seasonal migrations, constraints on migration behavior and den site fidelity may be limiting connectivity between complexes of dens across the landscape (Harvey, 2015). Adult males of this species in BC have been shown to travel a maximum of ~4 km from their den of origin prior to returning to hibernate, although migration behavior is not necessarily consistent among individual snakes or populations within the same geographical region (Harvey, 2015). Migration distances vary considerably between snakes that occupy different dens, even those neighboring one another (Gomez et al., 2015). Differences in migration patterns could potentially be a result of differences in prey availability, thermoregulatory opportunity, and habitat suitability, all of which are key factors for snake persistence (Lomas, Larsen, & Bishop, 2015). In the present study, straight‐line distances between den complexes defined across the landscape ranged from 15 km (within cluster) to 412 km (among cluster) (Table 2), distances well beyond that undertaken by an individual snake in a single season. Therefore, it could take several years for snakes to encounter those from other den complexes. Moreover, environmental variability among sampled regions across the range may also be decreasing the likelihood that individuals will disperse far enough to find mates from dens outside of a given den complex. Based upon migration estimates, this seems plausible; however, as we were unable to sample from all known dens across the distribution, our sampling at the targeted den complexes may have excluded potential intermediary populations that could provide connectivity.

Estimates of relatedness within and among den complexes also indicated that the behavior of these snakes may influence levels of migration and population connectivity. Snakes sampled within a given den complex were found to be more related than snakes among complexes within a geographic region. At a finer level, snakes sampled at the same den were found, in some instances, to be more related to one another than snakes across dens within a given den complex. This pattern of relatedness suggests that snakes are denning near related individuals and may be mating locally with snakes from nearby dens, while exhibiting some form of kin recognition to avoid inbreeding. Kin discrimination behavior has been previously demonstrated in captively raised Timber Rattlesnakes (*Crotalus horridus*; Clark, 2004; Clark, Brown, Stechert, & Zamudio, 2008), but is suspected to occur in most other pit viper species including the Cottonmouth (Hoss, Deutschman, Booth, & Clark, 2015).

In addition to behavioral constraints on snake movement, our results also provide varying evidence for barriers to gene flow among den complexes. There was support for barriers to gene flow among den complexes identified in the TNI region. High and significant values of pairwise differentiation among den complexes (Table 2), as well as no significant pattern of isolation‐by‐distance among dens within this region, are indicative of reduced population connectivity due to factors besides geographic distance. Previous studies of other snake species have found that roads not only alter snake movement and behavior (Andrews & Gibbons, 2005; Shepard, Kuhns, Dreslik, & Phillips, 2008), but also serve as barriers to gene flow. For example, roads with varying traffic volumes have been found to limit gene flow and connectivity among dens of Timber Rattlesnakes (Clark, Brown, Stechert, & Zamudio, 2010) as well as among subpopulations of Western Diamondback Rattlesnakes (Herrmann, Pozarowski, Ochoa, & Schuett, 2017). Roads have been further shown to impact connectivity within and among regional populations of Eastern Massasauga Rattlesnakes (*Sistrurus catenatus*) in Ontario, Canada (DiLeo, Rouse, Dávila, & Lougheed, 2013), where bodies of water were also found to affect patterns of population structure. Within the TNI region, the TNI‐W, TNI‐C, and TNI‐E den complexes are separated by major highways such as Highway 97, Highway 1, and Highway 5, as well as the Thompson River, Kamloops Lake, and extensive human development. Consequently, our findings of significant genetic structure and limited population connectivity in this region may be a result of the roads and bodies of water present among the den complexes that were sampled, in addition to the geographic distances between these groups. More spatially explicit analyses that examine patterns of isolation‐by‐resistance (McRae, 2006) could further parse the influence of roads, water bodies, and other landscape features on patterns of genetic differentiation and connectivity in this area.

In contrast to the TNI region, our data provided weak evidence for distinct geographical or anthropogenic barriers to gene flow within the VER, OKS, and WA regions. Despite significant *θ* estimates between den complexes within these regions (Table 2), all values were low and fall within the level of “inbreeding connectivity” (*θ* < 0.20) as defined by Lowe and Allendorf (2010). This distinction suggests that gene flow may be occurring between den complexes in each of these regions at a level that can counteract the negative effects of inbreeding (Lowe & Allendorf, 2010). Within OKS, significant migration was detected between the OS and WL den complexes, which is consistent with the low estimate of pairwise differentiation. Significant patterns of isolation‐by‐distance were also detected among dens in both OKS and WA, suggesting that geographic distance may be the main factor responsible for limiting population connectivity throughout these regions.

Consistent with previous predictions of connectivity between *C. oreganus* in Canada and populations further south in the United States (Stebbins, 2003), a significant level of gene flow was detected among the OKS and WA regions, specifically between the OS and WA‐S den complexes. Given that the OS den complex is closer in proximity to the WA‐N den complex, we expected to find that more migration would be occurring between these areas. While further investigation found a significant pattern of isolation‐by‐distance among dens across OS and WA‐*N* (*r* = .518, *p* = .002), other factors may be contributing to the genetic differentiation detected between these den complexes. In addition to geographic distance between these sites, there are several high‐elevation mountain peaks within the Pasayten Wilderness in Washington (USA) that are situated between OS and WA‐N, which may be inhibiting snake movement. In order for gene flow to occur between these groups, snakes would have to successfully traverse over these mountains at elevations >2,000 m, well above the elevation at which this species has been shown to occur in studies across its range in North America (Ashton, 2001; Gomez et al., 2015; Loughran, Beck, & Weaver, 2015; Macartney & Gregory, 1988; Maida et al., 2018; Winton et al., 2018). In contrast, elevation remains relatively low (< 834m) across the landscape from OS to WA‐S, potentially better facilitating connectivity between these two areas.

### 4.2 Genetic diversity and relatedness

Across both den complexes and individual dens, identified patterns of genetic variation aligned with the predictions of the central–marginal hypothesis for populations that exist at the periphery of a species distribution. Here, we saw reduced levels of genetic variation within the northernmost region of the Western Rattlesnake distribution, as evidenced by a significant negative relationship detected between latitude and within den complex estimates of nucleotide diversity (Figure 4). The proportion of polymorphism detected within den complexes also tended to be qualitatively lower at the northern extent of the species range in TNI and VER, and higher in the southern populations in OKS and WA (Table 5). Based upon inferred patterns of migration and genetic differentiation, it is likely that diversity measures are higher among den complexes within the OKS and WA regions due to inferred connectivity within and among these groups, which may extend further south into other populations of *C. oreganus* closer to the core of the range in North America. Future studies that include samples from rattlesnake populations at more southern latitudes would allow for more direct tests of the central–marginal hypothesis for this species to better understand how genetic diversity is distributed range‐wide.

Generally, observed levels of heterozygosity were either similar to or higher than those detected for populations of other vipers in North America using SNPs (Maigret, Cox, & Weisrock, 2020; Sovic, Fries, Martin, & Gibbs, 2019). Although not pervasive, almost all instances of significant heterozygote deficiency within Western Rattlesnake den complexes and individual dens were associated with significant levels of inbreeding (Table 4). The exceptions were the TNI‐W and WA‐S den complexes, where individual dens were not found to deviate from Hardy–Weinberg expectations or show evidence for significant levels of inbreeding. For the TNI‐W group, the reduced heterozygosity at the den complex level may be explained by the Wahlund effect (Wahlund, 1928), which results from the pooling of individuals from genetically structured groups, or in this case, dens. Further analysis of dens within this group revealed low, but significant, pairwise differentiation (*θ* = 0.0763; *p* < .001) between the TNI‐W01 and TNI‐W02 dens, suggesting that underlying substructure may be responsible for the reduced heterozygosity of the den complex as a whole. As for the WA‐S den complex, the only significant (*p* < .05) pairwise differentiation detected was between the WA‐S02 and WA‐S03 dens (*θ* = 0.0813). Rather than genetic substructure influencing heterozygosity at the den complex level in WA‐S, relatedness among sampled individuals may be leading to a “Family Wahlund Effect” (Castric, Bernatchez, Belkhir, & Bonhomme, 2002). In this den complex, mean pairwise relatedness was higher within den than among dens, suggesting that individual dens may be comprised of separate family groups. In fact, den WA‐03 contained three pairs of individuals with inferred relatedness at the level of half‐siblings (data not shown). As a result, the probability of having sampled sets of closely related individuals across this entire den complex is higher than expected if individuals were randomly mating across dens, thereby decreasing the overall observed levels of heterozygosity for the complex. A similar pattern has been found in other vertebrate systems (Castric et al., 2002; Jensen, Tapia, Caccone, & Russello, 2015).

### 4.3 Implications for conservation

Taken together, our results provide valuable information for conservation and management of Western Rattlesnakes at their northern range limits, and for communal denning vipers elsewhere. Evidence of significant population differentiation and limited connectivity within and among the main geographic regions of Western Rattlesnake occurrence in Canada suggest that management of this species as a single conservation unit may not be adequate for maintaining genetic diversity and population connectivity range‐wide. In particular, the magnitude of genetic differentiation between TNI, VER, and other sampled populations across the range may warrant reconsideration of the current designation of a single DU for *C. oreganus* in Canada, although our results only provide evidence for population discreteness. Further evaluation of the evolutionary significance of populations of this species is necessary prior to a formal recommendation to change DU status. Future studies using larger scale, genome‐wide data would allow for the detection of candidate or “outlier” loci under selection; if detected and validated, these loci can be used to quantify adaptive genetic variation and better address the evolutionary significance criterion (Funk et al., 2012).

In addition to informing conservation unit designation, the results of this study can help guide management strategies for this and other species with similar life‐history traits. For snake populations that occur in fragmented habitat, re‐establishing connectivity may be challenging. Based upon the inferred patterns of hierarchical population structure and migration, the rehabilitation of fragmented landscapes at key locations may be the most effective approach to restoring population connectivity at local (i.e., the den complex level) and regional scales. The identification and subsequent preservation/restoration of key movement corridors and critical habitats can help to encourage connectivity, whether it facilitates individual movements or the gradual expansion of existing populations. However, this approach would be challenging in landscapes where land ownership is primarily private and where strong legislation does not regulate land use to favor species at risk conservation and recovery. Furthermore, increased public outreach and education will be necessary to minimize human–snake conflicts if these animals are to re‐establish in areas from which they have been extirpated due to development.

An alternative approach for managing populations separated by unsuitable habitat or more distinct barriers could be to artificially increase gene flow through translocation. For example, in the north and central OKS region near the City of Kelowna (Figure 1), we were unable to sample snakes because population densities were too low or absent at potential denning sites. This is likely due to the intensity of human activities and development that is preventing gene flow among snakes in this area. In such cases where barriers to natural movement will be exceedingly difficult to remove, translocation could help introduce novel genetic variation across these populations and provide greater resilience to stochasticity and ongoing anthropogenic development. Translocations have been shown to be successful for both Antiguan Racers (*Alsophis antiguae*; Daltry et al., 2017) and Adders (*Vipera berus*; Madsen, Ujvari, & Olsson, 2004), resulting in bolstered population sizes and increased genetic variation (Madsen et al., 2004). However, it is important to note that in most instances, translocations are ineffective, especially for snakes and other reptiles (Brown, Bishop, & Brooks, 2009; Germano & Bishop, 2009; Nowak, Hare, & McNally, 2002; Reinert & Rupert, 1999). Many studies have found translocation to alter snake behavior and movement patterns, while also increasing population mortality (Reinert, 1991; Sullivan, Nowak, & Kwiatkowski, 2015). Additionally, the movement of individuals may also result in reduced genetic diversity for both newly established and source populations (Furlan et al., 2020), as well as outbreeding depression (Weeks et al., 2011). Due to the risks associated with translocations, it is imperative that they are biologically informed to maximize potential success. Several studies discuss the impacts of translocation on both population genetics and demography, and outline guidelines relevant to their use as a conservation tool (Furlan et al., 2020; Weeks et al., 2011). Notably, translocation efforts must account for species evolutionary trajectory as well as life‐history characteristics in order to help meet conservation goals.

Overall, the continued genetic monitoring of Western Rattlesnakes in this system can allow conservation managers to track changes in genetic diversity and population connectivity over time, as well as evaluate the outcomes of management efforts. The genotyping method employed in this study (GT‐seq, Campbell et al., 2015) is a reliable, quick, and relatively inexpensive approach for simultaneously genotyping hundreds to thousands of individuals at hundreds of SNPs, and can be successfully applied to minimally invasive DNA samples (Schmidt et al., 2020). As the environment is continually modified due to anthropogenic development and changing climates, leading‐edge genomic tools can allow wildlife and conservation managers to more effectively survey natural populations and adapt conservation strategies as necessary to encourage the persistence of species amidst their current decline.

## CONFLICT OF INTEREST

None declared.

## AUTHOR CONTRIBUTIONS

**Danielle A. Schmidt:** Conceptualization (supporting); Data curation (lead); Formal analysis (lead); Investigation (lead); Methodology (equal); Writing‐original draft (lead); Writing‐review & editing (equal). **Purnima Govindarajulu:** Conceptualization (supporting); Funding acquisition (equal); Resources (supporting); Writing‐review & editing (supporting). **Karl W. Larsen:** Conceptualization (supporting); Data curation (supporting); Funding acquisition (equal); Resources (supporting); Supervision (supporting); Writing‐review & editing (supporting). **Michael A. Russello:** Conceptualization (lead); Formal analysis (supporting); Funding acquisition (equal); Investigation (supporting); Methodology (supporting); Project administration (lead); Resources (equal); Supervision (lead); Writing‐original draft (supporting); Writing‐review & editing (equal).

## Data Availability

Individual metadata and SNP genotypic data collected via GT‐seq are deposited in DRYAD (https://doi.org/10.5061/dryad.fbg79cns4). All Illumina raw reads are available from the NCBI sequence read archive (BioProject ID: PRJNA631230).
