# CORRIGENDUM

**DOI:** 10.1002/ece3.7780

**Published:** 2021-06-25

In the recent article by Schmidt et al. (2020), the authors inadvertently neglected to acknowledge Daniel Beck, Joey Chase, and J. D. Brooks (Central Washington University), who contributed samples that were invaluable to this study. The updated Acknowledgements section is as follows:
